# 异基因造血干细胞移植后合并重症肌无力2例报告并文献复习

**DOI:** 10.3760/cma.j.cn121090-20240311-00089

**Published:** 2024-10

**Authors:** 亚军 石, 英 汉, 晓菲 张, 瑞 葸, 海 白, 涛 吴

**Affiliations:** 中国人民解放军联勤保障部队第九四〇医院血液科，兰州 730050 Department of Hematology, the 940th Hospital of Joint Logistics Support Force of Chinese People's Liberation Amy, Lanzhou 730050, China

## Abstract

异基因造血干细胞移植后合并重症肌无力（MG）发病急，易快速进展，严重威胁移植后患者的生存。本文报道2例急性髓系白血病患者，行异基因造血干细胞移植后首发症状为气短，逐渐出现抬颈无力、呼吸困难，经乙酰胆碱受体抗体、新斯的明试验等检测，最终均诊断为MG。予溴吡斯的明、糖皮质激素或利妥昔单抗等治疗后病情均好转。

## Abstract (en)

The onset of myasthenia gravis（MG）after allogeneic hematopoietic stem cell transplantation（allo-HSCT）seriously threatens the survival of patients, since it is acute, and is prone to rapid progression. Two patients with acute myeloid leukemia（AML）, who had undergone allo-HSCT developed shortness of breath, and gradually developed cervical weakness and dyspnea. The acetylcholine receptor（AChR）antibody and neostigmine test enabled the diagnosis of MG. The condition of the patients improved after treatment with pyridostigmine bromide, glucocorticoids and rituximab.

异基因造血干细胞移植（allo-HSCT）后合并重症肌无力（Myasthenia gravis，MG）较为罕见[1]–[2]，国内外报道较少。MG是一种由自身抗体介导的神经肌肉接头传递障碍的自身免疫性疾病。MG早期症状不典型，病情进展快，严重影响allo-HSCT患者的生存[3]。本文报道2例allo-HSCT后并发MG患者的诊疗过程，以引起血液科医师的重视，为此类疾病的诊疗提供参考。

## 临床资料

例1，男，29岁，因“患白血病5年，移植后46个月余，气短乏力4 d”于2018年7月6日入院。患者2014年1月无明显诱因出现头晕、乏力、胸闷、气短，活动后加重。2014年2月出现间断高热，最高体温40 °C。2014年3月就诊于当地医院，查血常规：WBC 8.39×10^9^/L，HGB 40 g/L，PLT 4×10^9^/L，骨髓细胞形态学检查示急性髓系白血病（AML），转入我院。复查骨髓形态学：原始粒细胞比例74.8％，考虑AML-M_2_型。白血病免疫分型：原始、幼稚细胞占82.7％，表达CD34、CD38、CD123、CD117、CD7、CD64、CD33、CD13、HLA-DR，胞质髓过氧化酶表达阳性，明确诊断为AML-M_2_。2014年4月予IA（伊达比星+阿糖胞苷）方案诱导治疗达完全缓解（CR），5月予IA方案巩固治疗，大剂量阿糖胞苷（HD Ara-C）1.5 g每12 h 1次、第1～3天强化治疗，6月予IA方案化疗，7月、8月予HD Ara-C 1.5 g每12 h 1次、第1～3天化疗2个疗程，治疗期间复查骨髓形态达CR。2014年9月采用改良BuCy预处理方案（具体为：阿糖胞苷2 g·m^−2^·d^−1^，−9 d、−8 d；白消安0.8 mg/kg每6 h 1次，−7 d、−6 d、−5 d；环磷酰胺60 mg·kg^−1^·d^−1^，−4 d、−3 d；司莫司汀250 mg/m^2^，−2 d），于2014年9月行亲缘HLA全相合异基因造血干细胞移植（姐供弟），供者年龄27岁。共输注单个核细胞（MNC）7.04×10^8^/kg，CD34^+^细胞6.8×10^6^/kg。移植后患者血象恢复良好，移植后规律复查，骨髓形态达CR，微小残留病（MRD）阴性，嵌合体呈完全嵌合状态。移植后30 d患者腹部出现暗红色斑疹，略突出皮肤表面，考虑皮肤急性移植物抗宿主病（aGVHD）Ⅱ级，予甲泼尼龙治疗后斑疹逐渐消褪，病情平稳出院。患者于院外肤色逐渐变暗，皮疹面积较在医院时扩大，耳后、颈部、前胸、后背、臀部、大腿均出现暗红色皮疹，2014年10月患者出现巩膜、皮肤黄染，颜面部水肿，小便为暗红色，肝功能：AST 147 IU/L，ALT 425 IU/L，总蛋白57.3 g/L，白蛋白37.5 g/L，球蛋白19.8 g/L，总胆红素120.6 µmol/L，直接胆红素105.2 µmol/L，间接胆红素15.4 µmol/L，γ-GGT 1 750 IU/L，总胆汁酸111 µmol/L；尿常规：胆红素（++）；间接、直接抗人球蛋白试验阴性。上腹部MRI：肝内胆管炎（轻度）；慢性胆囊炎急性发作。诊断为aGVHD Ⅲ度（皮肤、肝脏），予保肝、退黄、环孢素A联合他克莫司等治疗，患者黄疸消退不明显，予血浆置换，患者仍觉乏力，黄疸未消退，移植后88 d予保肝、退黄、环孢素A、他克莫司、甲泼尼龙等治疗并输注脐带间充质干细胞，黄疸较前消退后出院。院外甲泼尼龙、环孢素A逐渐减量，复查肝功能：胆红素基本正常。2015年1月查肝功能：AST 76 IU/L，ALT 168 IU/L，总蛋白49.5 g/L，球蛋白13.5 g/L，总胆红素29.20 µmol/L，直接胆红素20.40 µmol/L，γ-GGT 711 IU/L，总胆汁酸21 µmol/L；尿常规：尿胆原+；上腹部MRI示：左肾周、脾缘少许渗出，余未见确切异常。8月底及9月底再次出现巩膜黄染，尿色加深，考虑皮肤慢性移植物抗宿主病（cGVHD），行肺功能检查未见明显异常，予甲泼尼龙、他克莫司、脐带间充质干细胞、熊去氧胆酸、还原型谷胱甘肽等治疗，复查肝功能，ALT增高，<200 IU/L；胆红素正常。院外他克莫司及甲泼尼龙逐渐减量至停药。

患者于2018年6月着凉后出现乏力、头颈抬举困难，伴吞咽困难、口干、胸闷、烦躁及视力下降，上述症状夜间加重，无发热。患者2018年7月（移植后46个月余）上述症状进行性加重并出现呼吸困难，收治于重症医学科，予气管插管、呼吸机辅助呼吸，查体：浅昏迷，对疼痛刺激有肢体退缩反应，颈软、无抵抗，双侧面纹对称，双侧眼睑闭合不全、无力，四肢肌力Ⅳ级，双侧病理征阴性。行肌电图检查未见明显异常，乙酰胆碱受体抗体阳性，明确诊断为MG Ⅴ型。先后予静脉注射免疫球蛋白（IVIG）、溴吡斯的明（60 mg口服，每6 h 1次）、泼尼松（60 mg口服，每日1次）等治疗后，患者意识逐渐清楚，呼吸困难及吞咽困难明显减轻，拔除气管插管，可自主呼吸，抬颈逐渐有力，一般情况好转。治疗期间出现肺部感染，予美罗培南抗感染治疗，感染控制，于2018年8月出院。2023年12月复查，病情稳定。

例2，男，43岁，因“患白血病6年3个月余，移植后20个月余”于2019年5月16日入院。患者2017年2月无明显诱因出现右上肢散在出血点，此后皮肤出血点逐渐增多，4月于当地医院查血常规：WBC 8.3×10^9^/L，HGB 128 g/L，PLT 9×10^9^/L，考虑血小板减少，予地塞米松治疗，PLT上升不明显。2017年4月就诊于我科，骨髓形态学示原始粒细胞比例68.4％，提示AML-M_2a_；白血病免疫分型：可见明显异常细胞，占有核细胞比例3.5％，表达CD117、CD34、MPO、CD13、CD38、HLA-DR，为恶性髓系原始细胞；白血病43种融合基因筛查均阴性；AML预后基因提示：CCAAT增强子结合蛋白a（CEBPA）基因突变阳性，WT1基因/ABL1基因8.34％。染色体为正常核型。明确诊断为AML-M_2a_，2017年4月予IA方案化疗达CR，5月予TA（吡柔比星+阿糖胞苷）方案化疗，6月予阿糖胞苷2 g每12 h 1次，第1～3天化疗，7月予ME（米托蒽醌+依托泊苷）方案化疗。期间复查骨髓均达CR。2017年8月采用改良BuCy预处理方案（阿糖胞苷2 g·m^−2^·d^−1^，−10 d、−9 d；白消安0.8 mg/kg 每6 h 1次，−8 d、−7 d、−6 d；环磷酰胺1.8 g·m^−2^·d^−1^，−4 d、−3 d；司莫司汀250 mg/m^2^，−5 d）。患者于2017年9月行亲缘HLA全相合allo-HSCT（兄供弟），供者年龄44岁。共输注MNC 5.83×10^8^/kg，CD34^+^细胞3.47×10^6^/kg。移植后18 d复查骨髓形态学达CR，MRD阴性，嵌合体供者细胞占99.18％。移植后18 d患者出现颜面部、颈部、双手心及双足底皮肤潮红、红色皮疹，考虑皮肤aGVHD Ⅲ度，给予甲泼尼龙60 mg/d治疗后皮疹逐渐消褪，甲泼尼龙逐渐减量。移植后120 d出现多处关节疼痛，晨起加重，予塞来昔布等对症治疗后，上述症状缓解不佳，考虑cGVHD，予泼尼松30 mg/d治疗后疼痛缓解。患者于院外逐渐减停环孢素A及糖皮质激素。复查骨髓形态学达CR，嵌合体呈完全嵌合状态，CEBPA基因阴性。2018年2月患者再次出现颜面部皮疹，自行停用环孢素A及泼尼松，皮疹逐渐扩散至躯干，未予重视。患者逐渐出现眼干、口干、肛周不适，伴双手近端指间关节疼痛，晨僵，双踝关节肿胀，伴下午及夜间头痛，恶心，干呕，考虑cGVHD。2018年3月予泼尼松30 mg/d治疗，上述症状无改善，泼尼松加量至60 mg/d，期间患者口腔黏膜疼痛加重，联合芦可替尼（5 mg每日2次）治疗后，上述症状减轻出院。2018年5月查胸部CT：两肺间质性肺炎，较前为新发；两侧胸膜局限性增厚、粘连；双侧胸腔少量积液。予抗真菌、抗病毒及抗细菌等治疗后，复查胸部CT有所好转。持续服用芦可替尼5 mg每日2次，泼尼松10 mg每日2次，定期门诊复查，上述药物逐渐减量。此次患者于2019年5月（移植后20个月余）出现乏力，活动后加重，晨轻暮重，逐渐出现抬颈无力、气短，明显消瘦。入院后查血常规正常，肝功能：AST 112 IU/L，ALT 102 IU/L；自身抗体∶抗核抗体阳性（1∶80），抗核抗体主要核型为核仁型和胞浆颗粒型。复查骨髓形态学未见异常，MRD阴性，完全嵌合状态。胸部CT：双肺间质增生，无新发感染。行肺通气功能测定呈限制性中度减退，小气道功能测定呈阻塞性轻度减退。肌电图未见异常，新斯的明试验阳性。结合病史、临床表现、实验室检查等考虑MGⅢa型，予泼尼松30 mg/d治疗，血氧饱和度及呼吸频率均正常后出院，院外泼尼松逐渐加量至60 mg/d。此后颈部无力、气短改善，但容易反复，间断使用甲泼尼龙、IVIG、利妥昔单抗等免疫抑制治疗，气短、乏力减轻，病情稳定。近期于2023年5月16日入院复查无特殊不适，骨髓形态学未见异常，MRD阴性，病情稳定。

## 讨论及文献复习

allo-HSCT后合并神经系统疾病是严重影响患者预后的重要因素之一[2],[4]–[5]。其中，移植后MG较为罕见，易迅速进展，出现肌无力危象，若诊治不及时，易导致患者死亡[6]–[7]。本文2例患者行allo-HSCT后1～4年内均出现全身无力、抬颈困难，其中1例出现肌无力危象，经乙酰胆碱受体（AChR）抗体、新斯的明试验等明确诊断为MG，经糖皮质激素、IVIG或利妥昔单抗等治疗，2例患者的肌力逐渐恢复，呼吸困难改善，预后良好。

MG是由AChR或肌肉特异性受体酪氨酸激酶（MuSK）等抗体在细胞、体液免疫等的参与下，激活补体，攻击突触后膜的AChR等受体，导致无法产生肌肉收缩需要的终板电位，最终引起骨骼肌无力[8]。MG的典型临床表现：眼睑下垂、复视、构音障碍（发音不清）、吞咽困难、头部下垂、上肢或下肢近端肌肉的波动性无力、容易疲劳，呈典型的“晨轻暮重”[9]。累及呼吸肌的患者可迅速出现呼吸困难甚至呼吸衰竭，即肌无力危象，严重威胁患者生命[10]。MG的诊断需同时符合以下两个条件：①具有典型的临床症状如波动性肌无力、眼睑下垂或“晨轻暮重”等；②血清抗AChR抗体阳性、药理学检查如甲硫酸新斯的明试验阳性、电生理学特点如出现波幅递减等，满足其一即可[7]。Tsutsumi等[11]统计了24例allo-HSCT后合并MG的患者，19例患者的AChR抗体阳性，仅2例患者的MuSK抗体阳性，且多见于40岁以下的女性MG患者，主要症状为上睑下垂，复视，颈、肩、球部肌或呼吸肌无力[12]。急性加重期推荐使用IVIG及血浆置换，其他治疗包括最常用的胆碱酯酶抑制剂溴吡斯的明、糖皮质激素、免疫抑制剂、靶向生物制剂利妥昔单抗、依库珠单抗[7]，自体造血干细胞移植（auto-HSCT）等[13]。但MuSK抗体阳性患者对治疗的反应通常小于AChR抗体阳性的患者，对溴吡斯的明通常无效，对免疫抑制剂如环孢素A、霉酚酸酯、他克莫司等反应良好[12]。

有研究报道，allo-HSCT后合并MG的发生率不到1％[1],[14]。Sheikh等[2]通过检索文献纳入1 049例造血干细胞移植患者，共21例患者诊断MG，其中19例患者为allo-HSCT，2例为auto-HSCT，发生率2％。本中心近10年320例allo-HSCT患者中只有2例合并MG，发生率0.63％，与文献报道一致。移植后MG的发病时间跨度较大，有研究报道MG出现在allo-HSCT后3～100个月，中位时间为24个月[15]。本研究中2例MG患者的发病时间在移植后1～4年，与上述报道一致。

有研究统计了24例者移植后合并MG血液病患，发现再生障碍性贫血和慢性髓性白血病（CML）患者最多见，其次是AML，MG更常发生在以女性作为供者的男性患者中。同时，在合并MG的患者中，供者多为亲缘，多发生于cGVHD，多为HLA不相合[11]。Tsutsumi等[11]报道了1例AML患者移植后出现皮肤、口腔、肝脏和肺cGVHD，于allo-HSCT后1年8个月逐渐出现眼睑下垂及四肢无力，血清AChR抗体阳性，诊断MG，予甲泼尼龙、IVIG治疗，症状明显减轻。既往文献报道，6例CML患者行allo-HSCT后3～60个月诊断为MG，其中5例均合并cGVHD，1例未报道是否出现cGVHD[16]–[21]。

有文献报道，MG与以下特定HLA位点相关：HLA-Cw1、Cw7、DR2、DR3、DQ2和B8[9],[22]。有研究报道，1例allo-HSCT后合并MG的患者HLA配型结果呈全相合：A 24:02 24:20，B 52:01 55:02，Cw 01:02 12:02，DR 09:01 15:02[11]。另1例移植后出现MG的患者HLA配型为A02；B04，B06，B59，B61；Cw01，Cw03；DR04，DR53；DQ04，供者为A24，A02；B04，B06，B59，B61；Cw01，Cw03；DR04，DR53；DQ04[21]，2例均包含Cw1位点，Cw1被认为是cGVHD相关MG的危险因素[23]。也有学者认为MG可能是cGVHD的一种表现形式或与cGVHD相关，在免疫抑制剂减量或停用后发病，原因是供者的免疫细胞攻击受者的神经肌肉接头[12]，引起AChR破坏，导致骨骼肌无力。本文报道的2例MG患者均存在cGVHD，有少部分病例未发生cGVHD[12]。Tsutsumi等[11]检测了移植后患者的冷冻血清样本，发现血清AChR抗体呈阴性，在MG发病前3个月才转为阳性，有研究认为可能与allo-HSCT后B淋巴细胞介导的体液免疫失调有关，表现为自身反应性B细胞过度激活，产生自身抗体，攻击AChR[24]–[25]，无法产生终板电位，导致MG发病。Kotani等[21]发现MG发病前1个月外周血OX40^+^CD4^+^ T细胞升高，外周血CD4^+^ T细胞表达OX40可能是cGVHD相关自身免疫性疾病的标志物。同时，在特发性MG患者中，10％～20％的MG患者合并胸腺瘤，约80％的患者合并胸腺体积增大[10]，但allo-HSCT后出现MG的患者没有合并胸腺瘤，上述结果表明，allo-HSCT后继发MG的病因可能不同于特发性MG。

综上所述，当移植后出现全身乏力、肌力下降、眼睑下垂或“晨轻暮重”现象，伴吞咽困难、呼吸困难等症状时，需高度警惕MG，尽早进行AChR抗体或MuSK抗体检测、新斯的明试验等，一旦确诊，应立即启动治疗。
